# QuickNGS elevates Next-Generation Sequencing data analysis to a new level of automation

**DOI:** 10.1186/s12864-015-1695-x

**Published:** 2015-07-01

**Authors:** Prerana Wagle, Miloš Nikolić, Peter Frommolt

**Affiliations:** Bioinformatics Core Facility, CECAD Research Center, University of Cologne, Joseph-Stelzmann-Str. 26, 50931 Cologne, Germany; Center for Molecular Medicine, University of Cologne, Robert-Koch-Str. 21, Cologne, 50931 Germany

**Keywords:** Next-Generation Sequencing, Batch processing, Data management, High-performance computing, Analysis workflow

## Abstract

**Background:**

Next-Generation Sequencing (NGS) has emerged as a widely used tool in molecular biology. While time and cost for the sequencing itself are decreasing, the analysis of the massive amounts of data remains challenging. Since multiple algorithmic approaches for the basic data analysis have been developed, there is now an increasing need to efficiently use these tools to obtain results in reasonable time.

**Results:**

We have developed QuickNGS, a new workflow system for laboratories with the need to analyze data from multiple NGS projects at a time. QuickNGS takes advantage of parallel computing resources, a comprehensive back-end database, and a careful selection of previously published algorithmic approaches to build fully automated data analysis workflows. We demonstrate the efficiency of our new software by a comprehensive analysis of 10 RNA-Seq samples which we can finish in only a few minutes of hands-on time. The approach we have taken is suitable to process even much larger numbers of samples and multiple projects at a time.

**Conclusion:**

Our approach considerably reduces the barriers that still limit the usability of the powerful NGS technology and finally decreases the time to be spent before proceeding to further downstream analysis and interpretation of the data.

**Electronic supplementary material:**

The online version of this article (doi:10.1186/s12864-015-1695-x) contains supplementary material, which is available to authorized users.

## Background

Next-Generation Sequencing (NGS) has become the method of choice for molecular genetic analyses such as transcriptome profiling (RNA-Seq, miRNA-Seq), chromatin immunoprecipitation followed by sequencing (ChIP-Seq) as well as resequencing of complete genomes. Numerous software packages designed to analyze massive amounts of NGS data are now publicly available. Preprocessing of NGS data typically takes advantage of a complex hardware architecture composed of, for instance, a parallel compute cluster, a database server and a web server. As this requires specialized IT skills, the widespread access to NGS technology is still hampered by technical barriers. The primary data analysis is therefore often centralized into core laboratories which face the challenge of using a reasonable selection out of the available software packages to process a growing flow of newly produced data.

We introduce a new framework named QuickNGS which can be operated by IT staff in bioinformatics core labs to process vast amounts of data provided by their end users, typically experimental scientists. QuickNGS enables a major decrease in time and effort put into the primary analysis of NGS data, thus contributing to the further evolution of NGS into a standard tool with high accessibility to researchers.

## Implementation

**Fig. 1:**
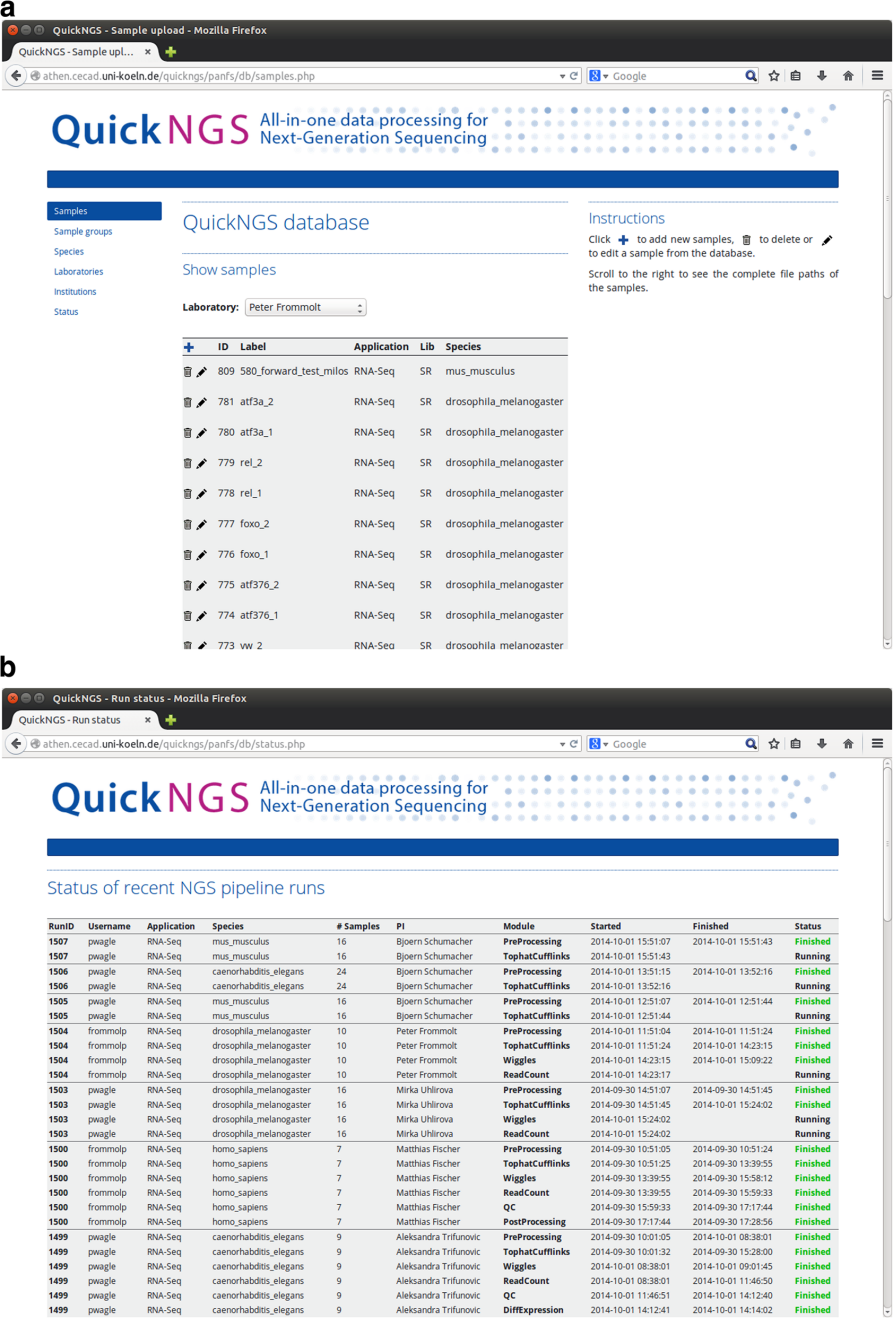
**a** The QuickNGS database contains meta information on samples (species, application, file locations, sample labels, lab name, library type, batch information) and sample groups (samples which are forming groups to be compared). Both can be efficiently organized by an intuitive web interface. New samples and sample groups can be inserted (Additional file 4) by following the „ + “button. **b** The status page on the QuickNGS database monitors time, user information and current status of each QuickNGS module on a clearly arranged website, enabling password-protected interrogation of the current status from any working location, including mobile access

**Fig. 2:**
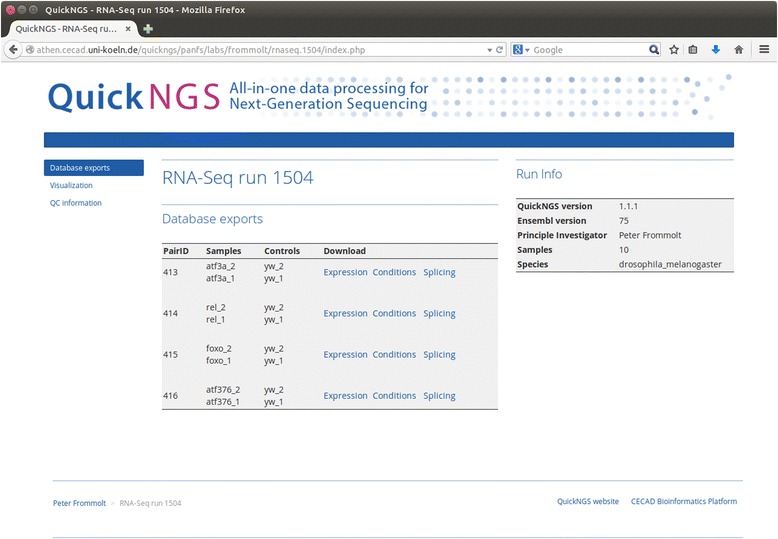
Results report for the test run on 10 Drosophila RNA-Seq samples. After login, the interface provides links to the main database export files, QC statistics as well as visualisation plots and the user’s personal password-protected UCSC genome browser track hub

QuickNGS enables rapid and professional data analysis for the aforementioned major applications of NGS in a batch-like operation mode. The core of the QuickNGS workflow is formed by a comprehensive MySQL database used to organize sample metadata such as species, library type and batch information as well as the analysis results. The system operators can use the QuickNGS *database interface* (Fig. 1a) to upload metadata and monitor the status of QuickNGS analysis runs from any location (Fig.1b). On the other hand, the purpose of the QuickNGS *web interface* is to deliver web reports on the analysis results to the end users who can use their personal login and password to browse the results plus a lot of useful on-top information (Fig. 2). The core results are provided as spreadsheet files which the user can download to a local computer.

As the sample metadata in the QuickNGS database are used to completely control the overall workflow, these have to be provided to the QuickNGS database before any analysis can be started. To achieve this, the file locations of the raw data first need to be saved into a text file (Additional file 4a). This file is then uploaded into the QuickNGS web interface (Additional file 4b) together with information on the library type, NGS application type, species of origin and the laboratory in which the input material has been generated. For each sample listed in the text file, the user is then asked to provide a human-readable sample label as well as batch information for the case that samples have been processed with different library preparation protocols or in different NGS runs (Additional file 4c). Finally, samples can be assigned to pairs and groups for comparative analysis, e.g. differential gene expression (Additional file 4d). Subsequently, the raw data received from the sequencing center are linked into the QuickNGS *stack directory* which is then processed fully automatically. Thus, the only user action needed is providing sample information and linking the files to the stack directory which both are trivial amounts of effort. The results produced by the workflow are saved back into the QuickNGS database and made accessible through a report on the web interface. This report comprises standard QC metrics (read counts, read quality, contamination, library quality, QC plots, cluster analyses etc.) and results on typical data-related research questions, for instance which genes are differentially expressed or differentially spliced, which genomic variations are unique to a sample compared to a control, which transcription factor binding motifs are enriched in a ChIP-Seq data set etc.. The results and report are generated fully automatically without any additional user action.

The analysis relies on widely adopted NGS analysis packages which are listed in Table 1. For the core analysis of the raw data, we have carefully selected the most appropriate previously published software programs. The selection criteria were (1) performance in published and in-house benchmarking studies, (2) comprehensiveness of the analysis output, (3) quality of the implementation and steadiness of maintenance, and (4) popularity in the community. Our choice of bioinformatics software follows these criteria as far as possible. The code for QC and visualisation as well as for data management and the workflow itself is unique to QuickNGS. As a reference to genomic sequence and annotation, the system uses the miRBase [8] for the miRNA-Seq workflow and the Ensembl database [9] for all other applications. For instance, RNA-Seq or ChIP-Seq analyses can thus be carried out on data from any arbitrary organism listed on either Ensembl (69 species as of release 76) or EnsemblGenomes (54 metazoa, 38 plants, 52 fungi, 32 protists, 15270 bacteria as of release 23). The reference files are downloaded to the local system and updated automatically. The same applies to genomic annotation data which are retrieved using BioMart [4].

**Table 1:** Algorithms and software tools used by QuickNGS, version 1.1.0 . The selection may be modified, updated or extended in future releases of QuickNGS, however, an up-to-date version of this table will be kept available online at the QuickNGS website

	Tool	Version	Reference
RNA-Seq	FastQC	0.10.1	
	Tophat2	2.0.10	[12]
	Cufflinks2	2.1.1	[19]
	DESeq2	1.4.5	[1]
	DEXSeq	1.10.5	[2]
	UCSC Genome Browser		[11]
miRNA-Seq	FastQC	0.10.1	
	miRDeep2	0.0.5	[6]
	DESeq2	1.4.5	[1]
	UCSC Genome Browser		[11]
ChIP-Seq	FastQC	0.10.1	
	BWA	0.7.7	[13]
	MACS2	2.0.10	[5]
	MEME-ChIP	4.10.0	[15]
	UCSC Genome Browser		[11]
WGS	FastQC	0.10.1	
	BWA	0.7.7	[13]
	Samtools	0.1.19	[13]
	Delly	2.0.1	[16]
	SnpEff	3.4	[3]
	UCSC Genome Browser		[11]

## Results and discussion

### Test run on previously published RNA-Seq data

**Fig. 3:**
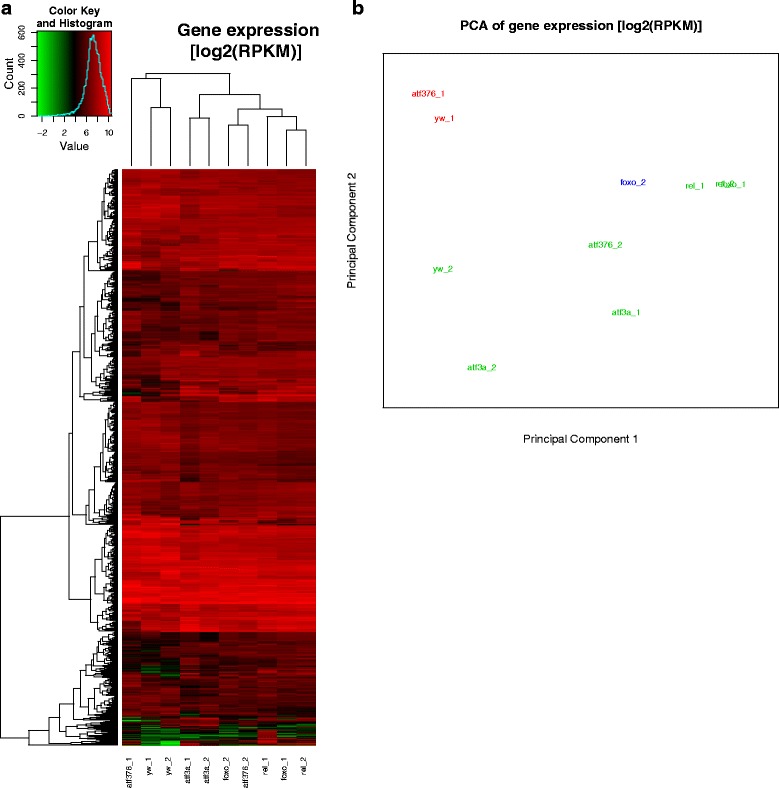
**a** Heatmap on the 10 RNA-Seq test data sets: The replicates of each genotype do not perfectly cluster together in distinct subclusters. This is likely to be cause by a combined effect of ribosomal contamination and batch effects. **b** The principle component analysis confirms that two samples (depicted in red) which were processed in a separate batch and with ribozero treatment cluster distantly from the remaining samples

To illustrate the practical use of our software, we have re-analyzed 10 RNA-Seq samples from a study on transcription factors in Drosophila where 4 mutant conditions were compared to a control with two biological replicates [18]. After feeding the sample metadata into the QuickNGS database, we have linked the 20 FastQ files into the QuickNGS stack directory. These preparing steps took us an overall time of 2 min. While waiting for the subsequent pipeline run to finish, we were able to monitor the current status of the respective modules using the status page on the QuickNGS database interface (Fig. 1b). The RNA-Seq workflow comprises an initial quality check using FastQC plus some software which is unique to QuickNGS. The basic data processing consists of a splicing-aware alignment using Tophat2 [12] followed by reference-guided transcriptome reassembly with Cufflinks2 [19]. Differential gene expression and differential exon usage are analyzed with DESeq2 ([1], Genome Biol) and DEXSeq ([2], Genome Res). After the processing finished overnight, we logged in to the QuickNGS user interface and found a report which summarizes all results of the QuickNGS workflow (Fig. 2). From the initial quality check, we received some basic read statistics (Table 2) as well as standard QC plots, a heat map (Fig. 3a) and a plot from a principle component analysis (Fig. 3b) for the 10 samples. The results of the core analysis for the comparison of atf3 mutants (atf3a_1 and atf3a_2) against controls (yw_1 and yw_2) are provided as Additional files. At thresholds 5 and 0.01 for fold-change and p-value, we get a set of 93 differentially expressed genes (Additional file 1) and a set of 168 differentially used exons (Additional file 2). Additional file 3 reports the p-values and fold-changes for differential gene expression (atf3a_1 and atf3a_2 compared to yw_1 and yw_2) together with those for the comparisons of the remaining three mutant conditions to control (atf376_1 and atf376_2, foxo_1 and foxo_2, rel_1 and rel_2, each compared against yw_1 and yw_2). On the web interface, the same three spreadsheet files are given also for these comparisons. All tables contain a comprehensive selection of genomic and functional annotation. Visualisation of the RNA-Seq wiggle files on the UCSC Genome Browser can be accessed by a hyperlink which uses a local password-protected track hub for the browser. The FastQ files for these test data are available from the NCBI Short Read Archive (SRA) at accession number SRP011390.

**Table 2:** Reads statistics on the test data from [18] – read counts are given in multiples of 10^6^ (1 M = 1 million reads). Duplicate removal was not performed because this was a single-read analysis. Two samples (yw_1 and atf376_1) were treated with ribo-zero, whereas for the remaining samples, there is a significant degree of contamination with ribosomal RNA. For all samples, about the half of the reads map to the original strand because all data origin from unstranded libraries

Label	# Reads	# Aligned	MapQ ≥ 30	Stranded	miRNA	rRNA	Other ncRNA
yw_1	25.9 M	17.4 M	16.2 M	50.2 %	0.0 M	0.3 M	0.1 M
yw_2	37.4 M	34.2 M	32.5 M	50.5 %	0.0 M	5.3 M	0.2 M
atf376_1	26.9 M	20.8 M	19.3 M	50.3 %	0.0 M	0.1 M	0.1 M
atf376_2	36.3 M	32.5 M	31.3 M	50.9 %	0.0 M	3.4 M	0.2 M
foxo_1	37.6 M	34.1 M	32.6 M	50.0 %	0.0 M	2.9 M	0.3 M
foxo_2	39.3 M	33.0 M	31.7 M	50.2 %	0.0 M	3.0 M	0.3 M
rel_1	37.8 M	34.6 M	32.9 M	50.0 %	0.0 M	2.9 M	0.3 M
rel_2	38.1 M	34.6 M	33.0 M	50.1 %	0.0 M	3.0 M	0.3 M
atf3a_1	38.4 M	34.7 M	33.2 M	50.3 %	0.0 M	3.8 M	0.3 M
atf3a_2	38.5 M	35.2 M	33.7 M	50.3 %	0.0 M	3.9 M	0.3 M

**Table 3:** Comparison of the technical features of QuickNGS to those of other NGS analysis workflow systems

	QuickNGS	Galaxy	GenePattern	Chipster
Setup	Compute cluster plus DB and web server	Client/server system	Client/server system	Client/server system
Applications	RNA-Seq, miRNA-Seq, ChIP-Seq, Whole-Genome	Universal framework	RNA-Seq	RNA-Seq, miRNA-Seq, ChIP-Seq, Whole-Genome
Database	Metadata and results	None	None	None
Workflow automation	Full	Started in web interface	Started in web interface	Started in client software
Reproducibility/Documentation	Results kept in DB Version tracking Logfiles	Workflow files	Workflow files	Workflow files
Workflow flexibility	Requires shell programming	Can be changed in web interface	Can be changed in web interface	Can be edited in client software
Purpose of user interface	End-user access to the analysis results	Data import and start of workflows	Data import and start of workflows	Data import and start of workflows

### Description of other QuickNGS workflows

Although the current QuickNGS release also comprises workflows for miRNA sequencing, ChIP-Seq and whole-genome resequencing, we gave above a detailed description only for the RNA-Seq workflow. However, the same level of efficiency and automation is also achieved in all other QuickNGS workflows. The miRNA-Seq workflow comprises quantification and differential profiling of 3p and 5p mature miRNAs using miRDeep [6] as well as statistics on miRNA families. Differential miRNA expression is profiled with the DESeq2 package [1]. The ChIP-Seq workflow takes advantage of BWA [14] for genomic alignment of the reads and uses MACS2 [5] for peak calling. Furthermore, QuickNGS identifies all genes which are 2000 bp up- or downstream from the MACS2 peaks. The peak sequences are analyzed for enrichment of transcription factor binding motifs using MEME-ChIP [15]. The results comprise lists of significant peaks and reports for motif enrichment. For the whole-genome resequencing workflow, finally, the software uses BWA for genomic alignment and calls single nucleotide polymorphisms with SAMtools [13] and structural variations with Delly [16]. The results are annotated and functionally classified with SNPeff [3]. Basic QC statistics and password-protected track hubs for the UCSC Genome Browser with direct hyperlinks for visualisation are part of all workflows. The QuickNGS database comes with ready-made metadata for additional test data which are available from the SRA at NCBI at accession numbers SRP043191 (miRNA-Seq), SRP007261 (ChIP-Seq) and SRP020555 (whole-genome resequencing). Additional modules dedicated to cancer genomics and more recent NGS applications such as CLIP-Seq (cross-linking immunoprecipitation followed by sequencing) are currently under development.

### Features of QuickNGS compared to other NGS workflow systems

In order to elaborate how QuickNGS performs in comparison to other NGS workflow systems, we discuss the features that are unique to our solution as well as its limitations. The degree of automation in QuickNGS is much higher than, for instance, that of an appropriate workflow in popular data analysis frameworks like Galaxy [7], GenePattern [17] or Chipster [10]. This makes the system more efficient for the typical standard analyses, but also less flexible to modifications. In particular, our system enables an extreme reduction of the hands-on (not computation) time that staff have to spend for the basic NGS data analyses. Data processing for tens or hundreds of samples can be initiated in less than 10 min. While the subsequent analyses completely run in the background, they can be monitored on the status website and, once finished, the results are ready for immediate access by any scientist without specific IT skills. In contrast to all other systems, the QuickNGS database is capable of organizing sample meta information along with the analysis results, enabling a high degree of reproducibility and documentation of what analyses have been done. This is essential whenever large numbers of samples are processed. Our software is also the only one to summarize all analysis results into user accounts with ready-to-deliver web reports. An overview of the features of several NGS workflow systems compared to QuickNGS is given in Table 3.

## Conclusions

We have contributed QuickNGS, a software which extremely reduces the efforts put into basic data processing for Next-Generation Sequencing. QuickNGS takes advantage of powerful hardware architectures and a comprehensive database to control the overall workflow. As a result, our approach enables laboratories with a high throughput of NGS data analyses to now accomplish their basic bioinformatics work on next-generation sequencing data essentially in zero time.

## Availability and requirements

Project home page: http://bifacility.uni-koeln.de/quickngs/web

Operating system: Linux

Programming languages: Bash scripting, Perl, R

Other requirements: MySQL server, Apache web server on separate machine

License: GNU GPL version 3

## Additional files

Additional file 1:
**List of genes differentially expressed between the atf3a mutants and the yw controls in our test dataset.** The fold-changes and p-values are produced from the DESeq2 package, whereas the FPKM values are taken from Cufflinks output. The file contains multiple tabs representing lists cut at particular thresholds for fold-change and p-value. Additional file 2:
**List of exons differentially used between the atf3a mutants and the yw controls according to the DEXSeq package.** The file contains multiple tabs representing lists cut at particular thresholds for fold-change and p-value. Additional file 3:
**List containing the same p-values and fold-changes as Additional file** **1** **plus p-values and fold-changes for all other comparisons performed in the current QuickNGS runs.** In this example, these are the comparisons of the three other mutant condition against wild type. This table facilitates comparisons between the genes differentially regulated under one condition and other conditions. Additional file 4:
**Procedure to upload sample meta data into the QuickNGS database.** (**a**) First, the file locations of the raw data need to be saved into a text file. (**b**) Together with information on library type, NGS application, species and laboratory, this file can be uploaded into the QuickNGS web interface. (**c**) Human-readable sample labels as well as batch information can be provided for each sample listed in the text file. (**d**) Pairs of samples or sample groups can be defined for comparative analysis within the workflow.

## Abbreviations

NGS: Next-Generation Sequencing
DB: Database
QC: Quality check
IT: Information technology
SQL: Structured query language
ChIP: Chromatin immunoprecipitation
