# Supplementary figures and images for: QuickNGS elevates Next-Generation Sequencing data analysis to a new level of automation
Source: BMC Genomics. 2015 Jul 1;16(1):487. doi: 10.1186/s12864-015-1695-x (PMC4486389; doi:10.1186/s12864-015-1695-x)

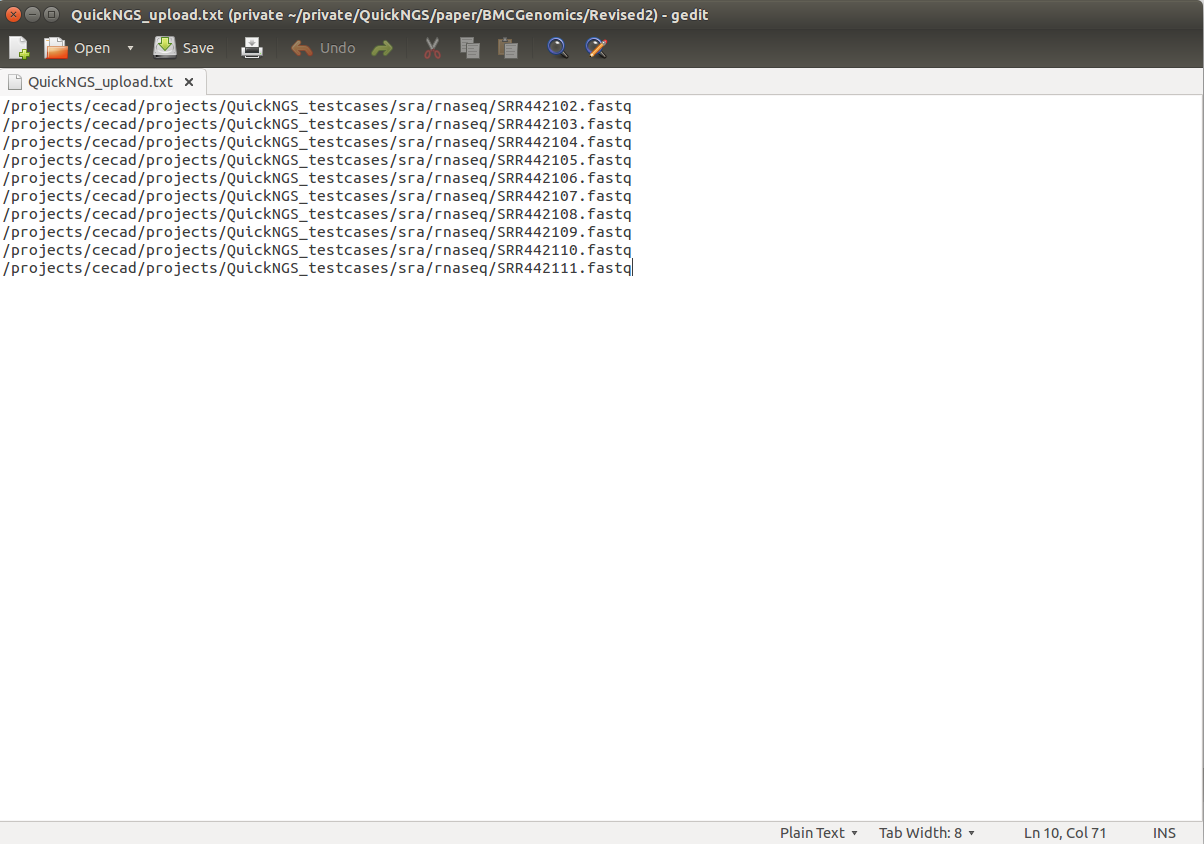

Supplement: Additional file 4: — Procedure to upload sample meta data into the QuickNGS database. (a) First, the file locations of the raw data need to be saved into a text file. (b) Together with information on library type, NGS application, species and laboratory, this file can be uploaded into the QuickNGS web interface. (c) Human-readable sample labels as well as batch information can be provided for each sample listed in the text file. (d) Pairs of samples or sample groups can be defined for comparative analysis within the workflow. [file 12864_2015_1695_MOESM4_ESM.zip › 5212076041449535_add4.png]

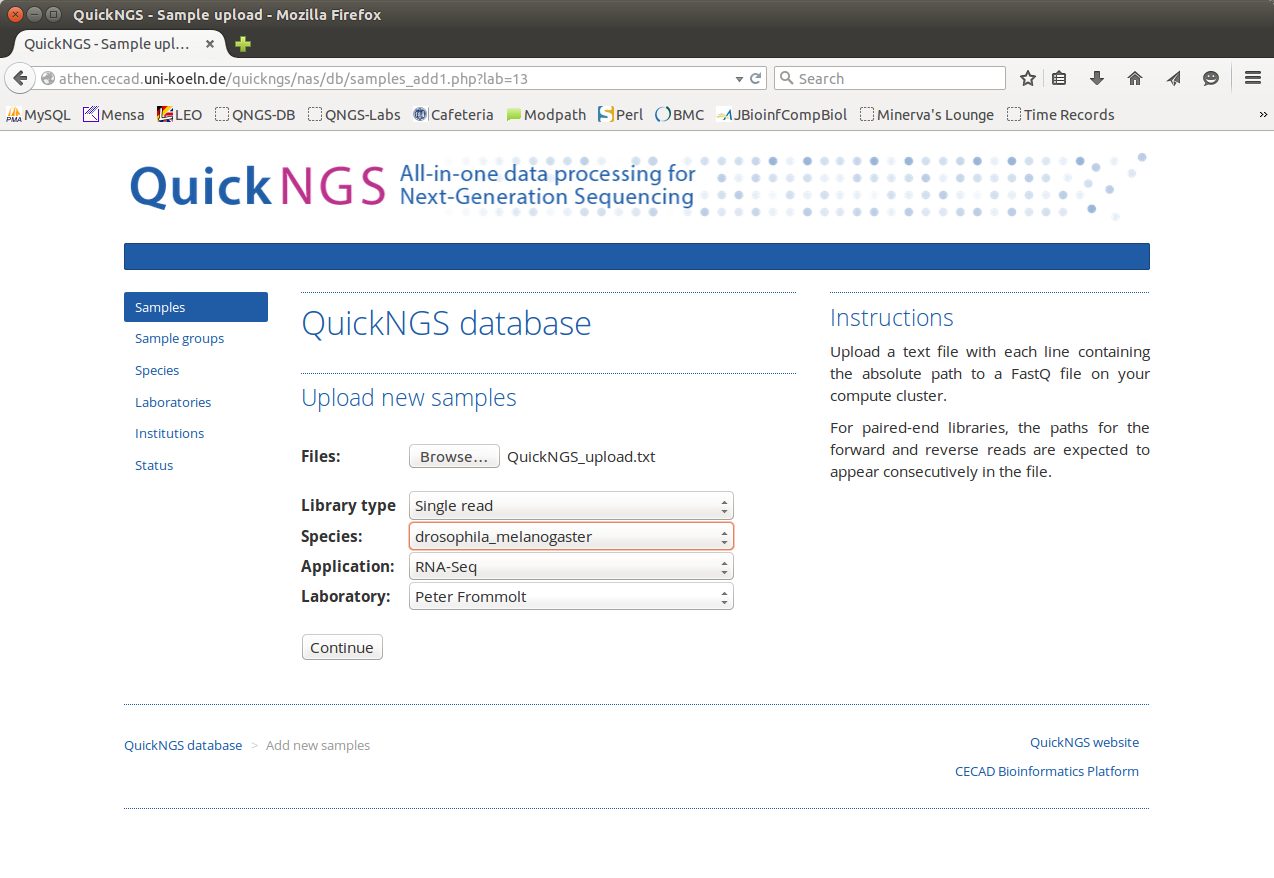

Supplement: Additional file 4: — Procedure to upload sample meta data into the QuickNGS database. (a) First, the file locations of the raw data need to be saved into a text file. (b) Together with information on library type, NGS application, species and laboratory, this file can be uploaded into the QuickNGS web interface. (c) Human-readable sample labels as well as batch information can be provided for each sample listed in the text file. (d) Pairs of samples or sample groups can be defined for comparative analysis within the workflow. [file 12864_2015_1695_MOESM4_ESM.zip › 5212076041449535_add5.png]

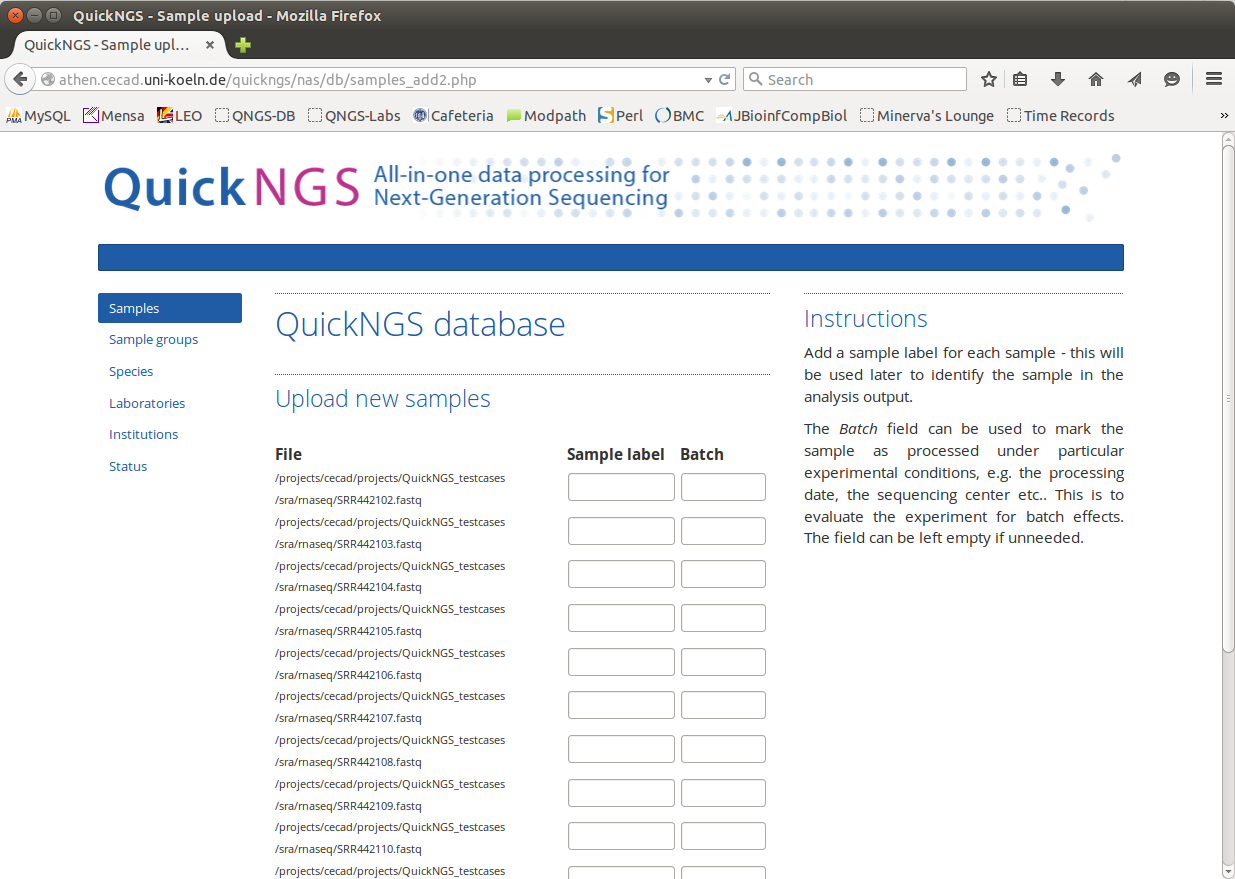

Supplement: Additional file 4: — Procedure to upload sample meta data into the QuickNGS database. (a) First, the file locations of the raw data need to be saved into a text file. (b) Together with information on library type, NGS application, species and laboratory, this file can be uploaded into the QuickNGS web interface. (c) Human-readable sample labels as well as batch information can be provided for each sample listed in the text file. (d) Pairs of samples or sample groups can be defined for comparative analysis within the workflow. [file 12864_2015_1695_MOESM4_ESM.zip › 5212076041449535_add6.png]

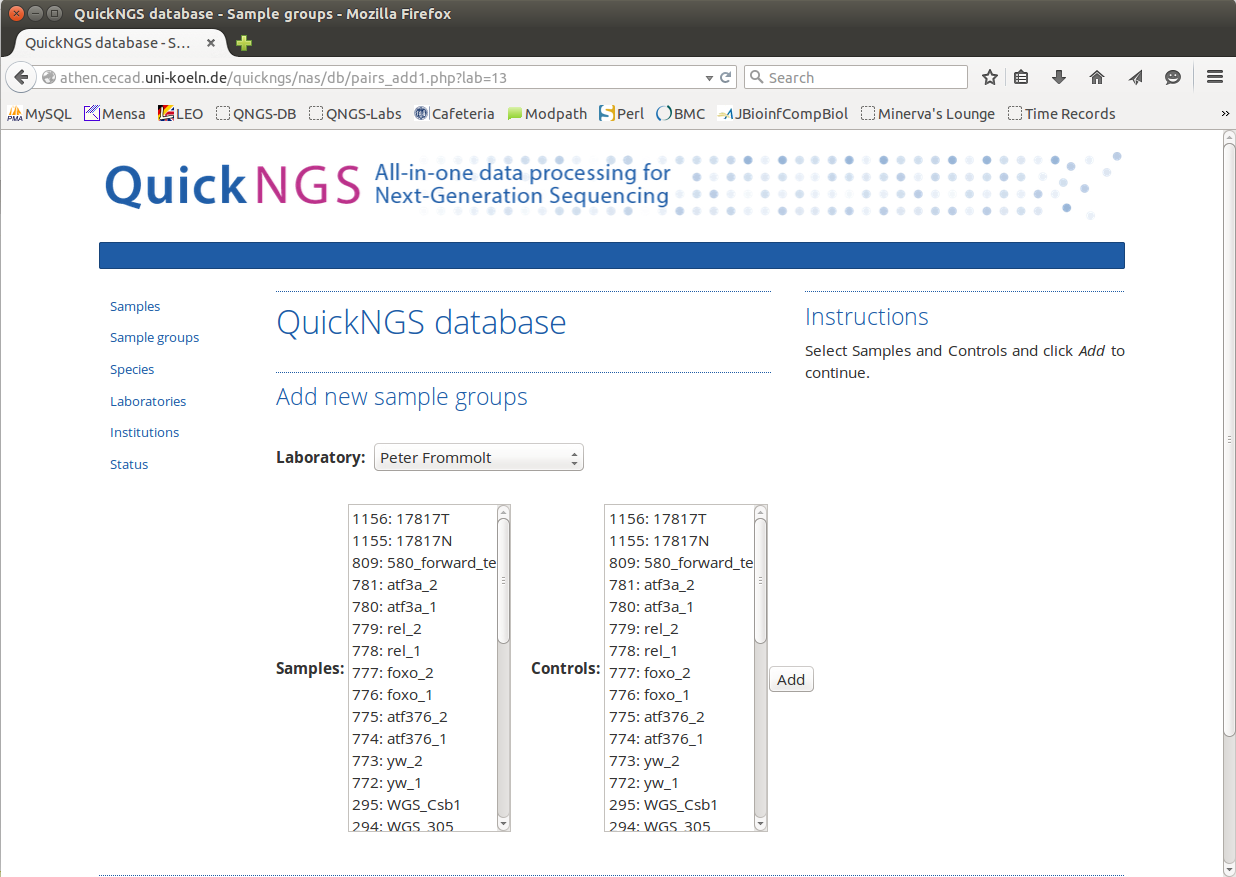

Supplement: Additional file 4: — Procedure to upload sample meta data into the QuickNGS database. (a) First, the file locations of the raw data need to be saved into a text file. (b) Together with information on library type, NGS application, species and laboratory, this file can be uploaded into the QuickNGS web interface. (c) Human-readable sample labels as well as batch information can be provided for each sample listed in the text file. (d) Pairs of samples or sample groups can be defined for comparative analysis within the workflow. [file 12864_2015_1695_MOESM4_ESM.zip › 5212076041449535_add7.png]
